# Supplementary material for: Role of the Ca2+-ATPase Pump (SERCA) in Capacitation and the Acrosome Reaction of Cryopreserved Bull Spermatozoa
Source: Cells. 2025 Nov 28;14(23):1892. doi: 10.3390/cells14231892 (PMC12691308; doi:10.3390/cells14231892)
Supplement: Supplementary file 1 [file cells-14-01892-s001.zip › SUPPLEMENTARY- TABLE S1-parameters of motility.pdf]

Time 0 min				
	Control	n	Treated	n
VSL	73,46±2.44	349	68,07±3.45	171
VCL	234,7±3.3	458	222,9±5.9	171
VAP	112,1±2.1	457	101,5±3.69	171
LIN	32,92 ± 0,6906	458	31,27 ±1,31	171
WOB	49,04 ± 0,6164	457	46,72 ±1,29	171
STR	66,92± 1,059	457	66,89± 1,901	171
ALH mean	2,946±0,05748	458	3,133±0,1222	171
ALH Max	5,292±0,1155	458	5,346±0,2335	171
BCF	27,45±0,432	457	25,68±0,7038	171
Dance	710,8±18,42	458	721,3±37,54	171
MAD	183,2±2,941	456	187,5±4,149	169
Factor ID	1,503±0,01153	458	1,546±0,02303	171

Time 10 min				
	Control	n	Treated	n
VSL	54,27±2,092	282	64,95±3,163	280
VCL	188,4±5,346	282	187,1±3,163	280
VAP	112,1± 2,095	282	101,5± 3,689	279
LIN	90,9±2,846	282	36,34±1,146	280
WOB	52,4±1,082	282	54,73±1,054	279
STR	63,94±1,443	282	66,16±1,523	279
ALH mean	2,531±0,1002	282	2,722±0,1031	280
ALH Max	2,222±0,09829	282	3,551±0,1926	280
BCF	19,95±0,6323	282	23,21±0,7759	279
Dance	559,8±35,32	282	563,8±30,42	280
MAD	167,5±3,804	280	182,1±3,858	276
Factor ID	1,503±0,01153	282	1,546±0,02303	280

Time 20 min				
	Control	n	Treated	n
VSL	56,78±2,463	179	40,42±2,442	70
VCL	162,4±5,949	179	148,1±8,092	70
VAP	90,9±2,846	179	97,3±3,291	70
LIN	40,6±1,593	179	30,7±1,852	70
WOB	57±1,547	179	56,98±2,37557	70
STR	70,66±1,617	179	56,98±2,375	70
ALH mean	2,222±0,09829	179	2,281±0,1088	70
ALH Max	5,292±0,1155	179	5,346±0,2335	70
BCF	18,49±0,9354	179	20,5±1,326	70
Dance	410,4±29,96	179	364,9±31,92	70
MAD	176±4,942	178	170,9±6,518	70
Factor ID	1,503±0,01153	179	1,546±0,02303	70

Time 30 min				
	Control	n	Treated	n
VSL	50,29±3,356	138	53,21±6,873	43
VCL	163±8,067	138	223,9±13,39	44
VAP	83,38±3,085	137	76,06±3,645	44
LIN	37,41± 1,81	138	27,64± 3,152	44
WOB	55,17± 1,714	137	55,11± 2,642	44
STR	67,65±2,53	137	51,12±4,127	44
ALH mean	1,975±0,1215	138	2,79±0,1758	44
ALH Max	3,167±0,2107	138	4,965±0,3665	44
BCF	18,4±1,001	137	27,03±1,767	44
Dance	386±33,95	138	646,6±64,28	44
MAD	149,8±4,884	137	183,1±8,223	43
Factor ID	1,503±0,01153	138	1,546±0,02303	44
