# Supplementary figures and images for: Role of the Ca2+-ATPase Pump (SERCA) in Capacitation and the Acrosome Reaction of Cryopreserved Bull Spermatozoa
Source: Cells. 2025 Nov 28;14(23):1892. doi: 10.3390/cells14231892 (PMC12691308; doi:10.3390/cells14231892)

Supplementary figure 3.

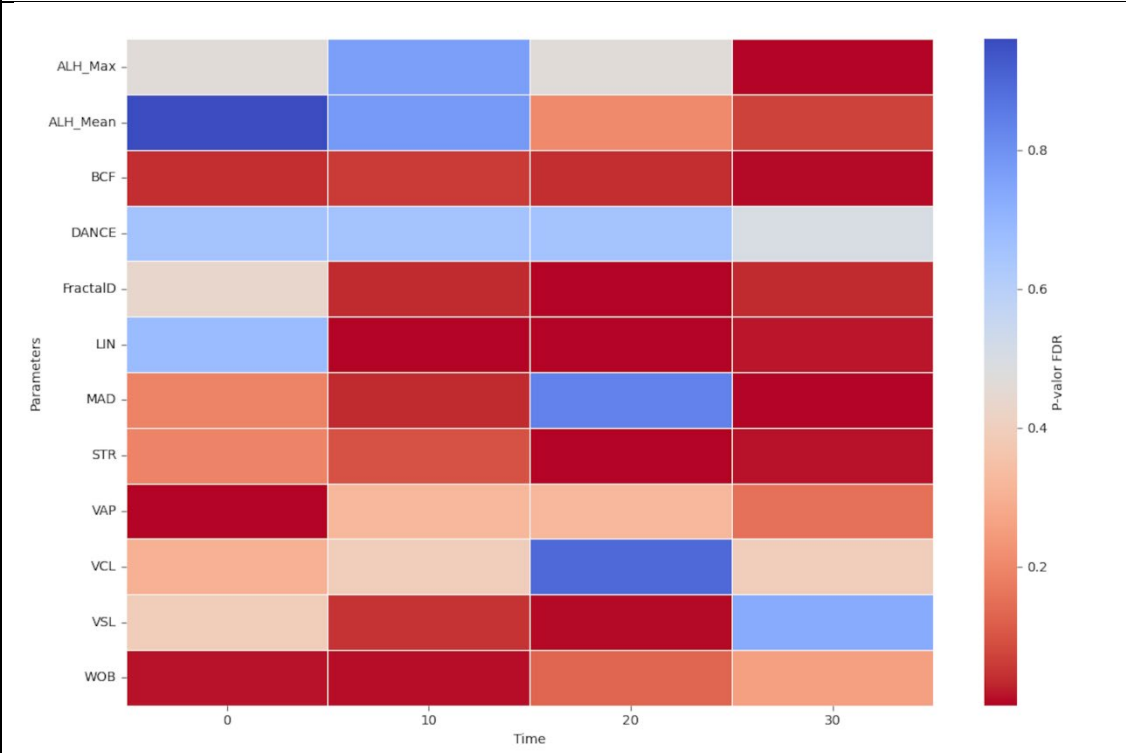

Supplement: Supplementary file 1 [file cells-14-01892-s001.zip › Supplementary figure S3.pdf]

Supplementary Figure 1.

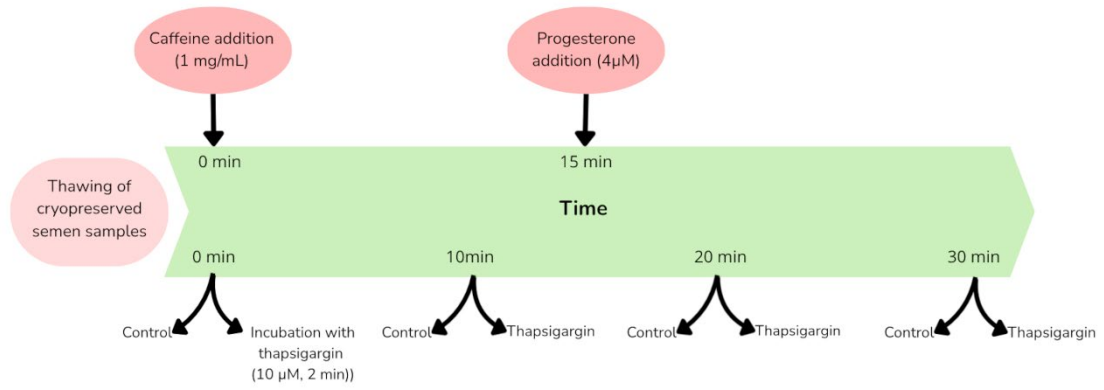

Supplement: Supplementary file 1 [file cells-14-01892-s001.zip › Supplementary figure-S1.pdf]

**Anti-SERCA**

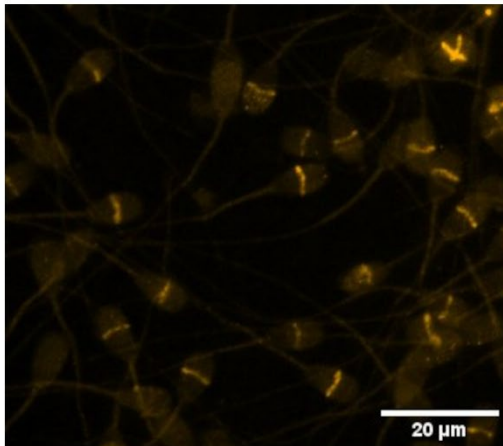

**Cy3 Control -**

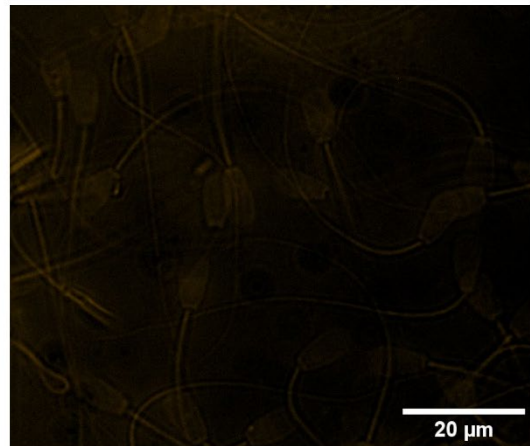

**Merge with transmitted light**

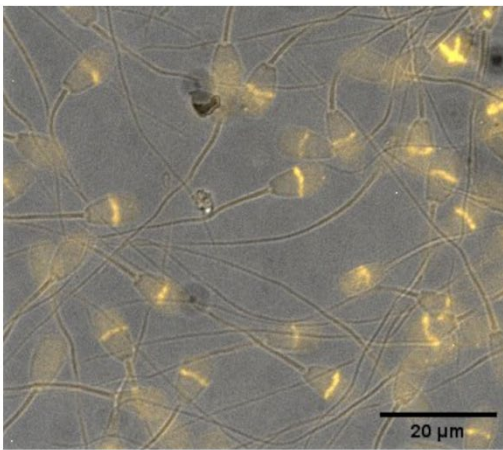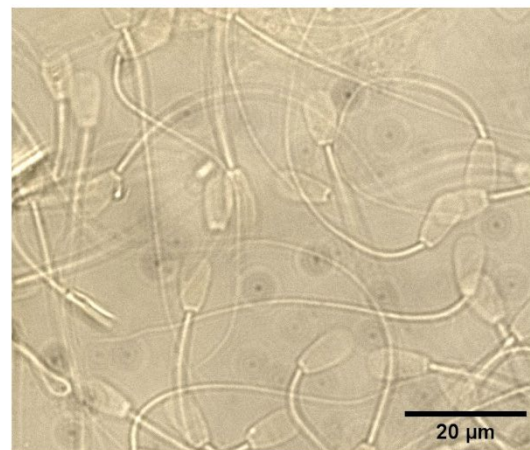

Supplementary figure 2.

Supplement: Supplementary file 1 [file cells-14-01892-s001.zip › Supplementary figure-S2.pdf]
